# Are D-*manno*-configured Amadori products ligands of the bacterial lectin FimH?

**DOI:** 10.3762/bjoc.11.123

**Published:** 2015-06-30

**Authors:** Tobias-Elias Gloe, Insa Stamer, Cornelia Hojnik, Tanja M Wrodnigg, Thisbe K Lindhorst

**Affiliations:** 1 Christiana Albertina University of Kiel, Otto Diels Institute of Organic Chemistry, Otto-Hahn-Platz 3/4, D-24118 Kiel, Germany, Fax: +49 431 8807410,; 2 Glycogroup, Institute of Organic Chemistry, Technical University Graz, Stremayrgasse 9, A-8010 Graz, Austria

**Keywords:** Amadori rearrangement, bacterial adhesion, *C*-mannosides, docking studies, FimH ligands

## Abstract

The Amadori rearrangement was employed for the synthesis of *C*-glycosyl-type D-mannoside analogues, namely 1-propargylamino- and 1-phenylamino-1-deoxy-α-D-*manno*-heptopyranose. They were investigated as ligands of type 1-fimbriated *E. coli* bacteria by means of molecular docking and bacterial adhesion studies. It turns out that Amadori rearrangement products have a limited activity as inhibitors of bacterial adhesion because the β-*C*-glycosidically linked aglycone considerably hampers complexation within the carbohydrate binding site of the type 1-fimbrial lectin FimH.

## Introduction

**Scheme 1:**
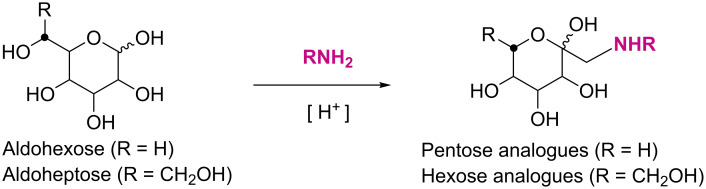
The Amadori rearrangement of aldoses with amines leads to *C*-glycosyl-type glycoconjugates, namely 1-amino-1-deoxyketoses.

The Amadori rearrangement (AR) is the reaction in which aldohexoses react with suitable amines under acidic catalysis to 1-amino-1-deoxyketohexoses (*C*-glycosyl-type pentose analogues) without the need of hydroxy group protection (Scheme 1). For a long time this reaction has been judged as unsuitable for preparative use as it typically leads to a complex mixture of products accompanied with a low yield of the rearrangement product itself [1]. However, we could show that the Amadori rearrangement, when applied to selected aldoses as starting materials, is a high yielding and efficient synthetic approach towards *C*-glycosyl-type glycoconjugates. For example, when aldoheptoses are employed as starting material for the Amadori rearrangement, the respective 1-amino-1-deoxyketoheptoses (*C*-glycosyl-type hexose analogues) can be obtained in exclusively one anomeric form as well as in excellent yields (Scheme 1) [2–3]. Thus, the Amadori rearrangement can be utilised to convert a respectively configured aldoheptose into a *C*-glycosyl-type glycoconjugate in one step and without the need of protecting group manipulations. This is intriguing in light of biorthogonal ligation methodology as Amadori products are structurally closely related to naturally occurring D-hexopyranosides. In addition, *C*-glycosyl glycoconjugates are believed to bear great potential as therapeutics and as tools for mechanistic studies in biology. This is because they are not sensitive towards enzymatic hydrolysis such as in physiological environment, in contrast to the naturally occurring *O*- and *N*-glycosides.

**Figure 1:**
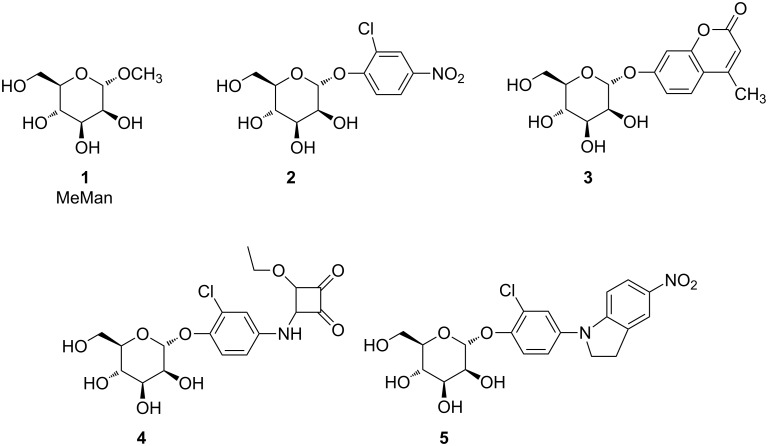
The bacterial lectin FimH is known to bind α-D-mannosides such as methyl α-D-mannoside **1** (MeMan) with IC_50_ values in the millimolar range [4]. Based on MeMan (**1**), the affinity of the *p*NPMan derivative **2** is 717-times improved [13], that of the methylumbelliferyl mannoside **3** 116 times improved [14], that of the squaric ester monoester **4** 6900 times higher [15], and the indolinylphenyl mannoside **5** arrives at an IC_50_ of 2.4 nM [8].

With respect to our long-lasting interest in the design and investigation of ligands for the bacterial lectin FimH [4] it has been our goal to investigate the Amadori rearrangement as a method to approach new FimH ligands. These are especially relevant in the context of an anti-adhesion therapy against bacterial infections [5–6]. As FimH-mediated adhesion to the glycosylated surface of host cells is a key step in infections caused by type 1-fimbriated bacteria, FimH antagonists that inhibit bacterial adhesion can be valuable for treatment of infectious diseases [7–8]. The structure of type 1-fimbrial lectin FimH has been elucidated in X-ray analysis [9–11]. Obviously, FimH binds α-D-mannosides such as simple methyl α-D-mannoside (MeMan, **1**) but not β-mannosides. Mannosides with an aromatic aglycone, such as *p*-nitrophenyl α-D-mannoside (*p*NPMan) and 4-methylumbelliferyl α-D-mannoside (**3**) show an improved affinity to FimH due to π–π-stacking interactions of the aromatic moiety with the so-called tyrosine gate at the entrance of the carbohydrate binding site, formed by Y48 and Y137. Additional interactions exerted by extended aglycone portions can further improve ligand affinity for FimH; for example *ortho*-chloro substitution of the phenyl ring (compounds **2** and **5**), a squaric acid partial structure (compound **4**) or heterocyclic substituents such as in indolinylphenyl mannoside **5** as recently introduced [12] (Figure 1).

With the structural requirements of the type 1-fimbrial lectin FimH for its ligands in mind, we addressed the question, if D-*manno*-configured Amadori products with their axially oriented anomeric hydroxy group can function as a new class of FimH ligands. In addition, we can assume that Amadori products are stable against cleavage by mannosidases, as we found earlier that D-*gluco*-configured Amadori products are no substrates for glucosidases.

## Results and Discussion

### Synthesis of heptopyranose **8** and Amadori products **9** and **10**

**Scheme 2:**
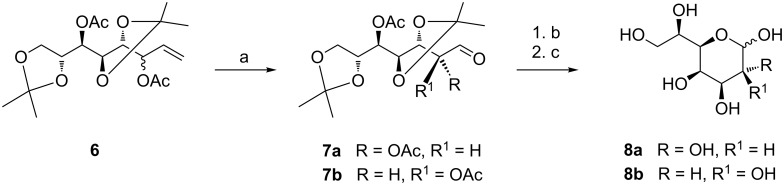
Synthesis of D-*glycero*-D-*galacto*/D-*talo*-heptopyranose **8a** and **8b**: a) O_3_, NaOAc, Me_2_S, CH_2_Cl_2_/MeOH, −50 °C, b) NaOMe, MeOH; c) ion exchange resin IR 120 H^+^, H_2_O/MeCN.

To access *manno*-configured rearrangement products for the synthesis of FimH ligands, we needed to synthesise the appropriate aldoheptose starting material. Its synthesis starts with the oct-1-enitol derivative **6** which can be easily obtained by a Grignard reaction of 2,3:5,6-di-*O*-isopropylidene-D-mannose employing commercially available vinylmagnesium bromide (Scheme 2) [16–17]. This C-elongation approach leads to a mixture of C-2 diastereomers, however, during the Amadori rearrangement this centre is converted to a keto group and thus separation of the C-2 epimeric mixture prior to the Amadori rearrangement is not necessary. Simple ozonolysis of the diastereomeric mixture **6** afforded a mixture of the protected D-*glycero*-D-*galacto*- and D-*glycero*-D-*talo*-configured heptoses **7a** and **7b** in quantitative yield. After sequential cleavage of the protecting groups, employing Zemplén conditions to remove acetyl groups [18–19] followed by acidic cleavage of the isopropylidene groups, the desired starting material for the Amadori rearrangement, a mixture of D-*glycero*-D-*galacto*/D-*talo* heptopyranoses (**8a/b**) was obtained in an overall yield of 85% from **6**. This is a synthetic route to aldoheptoses **8a** and **8b** alternative to the one reported [2] with the advantage that the use of environmental hazardous as well as highly toxic HCN is not required.

**Scheme 3:**
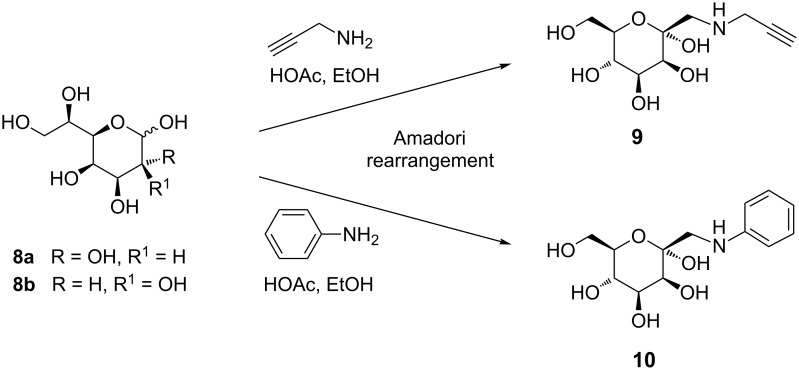
Amadori rearrangement of heptoaldose **8** with propargylamine and aniline to yield *C*-glycosyl-type D-mannoside derivatives **9** and **10**, respectively.

Amadori rearrangement of the diasteromeric mixture **8** with an amine of choice allows an efficient and versatile approach towards D-*manno*-configured *C*-glycosyl-type glycoconjugates. In our study, we have employed two different amines in the Amadori rearrangement with **8**, propargylamine and aniline. Under typical conditions for this reaction [2] 1-progargylamino-1-deoxy-D-*manno*-heptulose **9** and 1-phenylamino-1-deoxy-D-*manno*-heptulose **10** were obtained as pure α-anomers in 77% and 24% yield, respectively (Scheme 3). The low yield of compound **10** may be explained by the low p*K*_a_ value (4.62) of aniline compared to a p*K*_a_ of 8.15 for propargylamine, the latter being clearly more efficient as a nucleophile for this type of reaction. Analogous observations have been made in previous studies [20].

Rearrangement products **9** and **10** exist in their ^5^C_2_ pyranoid conformation as determined by NMR analysis and can thus indeed be regarded as analogues of α-D-mannosides. The *N*-alkyl/aryl aminomethyl substituent at the anomeric position is found in the sterically favoured equatorial position located towards the β-face of the sugar ring, whereas the anomeric hydroxy group is α-positioned. Whether this particular *C*-type glycoside architecture is suited for FimH complexation had to be tested.

### Theoretical consideration of Amadori products **9** and **10** as FimH ligands

**Figure 2:**
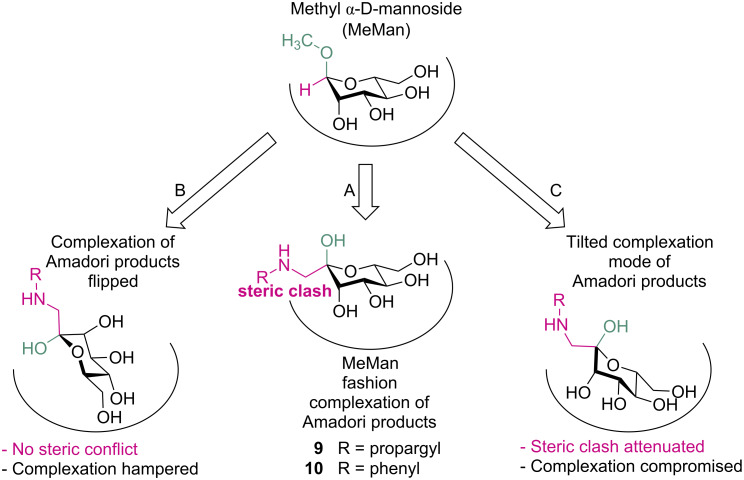
Cartoon illustrating ligand binding by the bacterial lectin FimH. Complexation of D-*manno*-configured *C*-glycosyl-type glycoconjugates inside the carbohydrate recognition domain (CRD) of FimH in analogy to MeMan (**1**, top) is hampered by the bulky *C*-glycosidic substituents in β-position, resulting in a steric clash (A). Rotation of the Amadori product averts steric conflict, yet causing insufficient complexation inside the binding pocket (B). Compromised complexation and attenuation of steric conflict may be achieved by a tilted orientation of the mannoside within the FimH carbohydrate binding site (C).

The complexation of MeMan (**1**, cf. Figure 1) as the most simple FimH ligand in the carbohydrate binding site of FimH has been described in detail [10]. It is depicted in a simplified cartoon fashion in Figure 2. The α-configured aglycone moiety (OCH_3_ in green) of the glycoside is pointing out of the binding site, whereas the axial 2-OH group as well as all other hydroxy groups of the sugar ring are complexed within the FimH carbohydrate binding site. Complexation of mannoside ligands is further supported by a conserved water molecule inside the carbohydrate binding site that is interacting mainly with the 2-OH group of the sugar ring. When the standard FimH ligand MeMan (**1**) is compared with the D-*manno*-configured *C*-glycosyl-type glycoconjugates **9** and **10**, emerging from Amadori rearrangement of the corresponding heptopyranose **8**, the axial methoxy moiety in MeMan (**1**) can be correlated with the equally axial oriented anomeric OH group of the Amadori products (Figure 2A). Then however, the equatorial anomeric (*N*-alkyl/aryl amino)methylene groups in **9** and **10** cause a steric clash in the binding pocket because of their bulkiness. To avoid this steric conflict, the Amadori products could be flipped such that the bulky aminomethyl substituent is pointing outwards of the sugar binding site (Figure 2B). But then, the anomeric hydroxy group might be sterically hindering. In addition, proper complexation of the sugar ring will be hampered due to considerable alteration of the 3D pattern of ring hydroxy groups available for hydrogen bonding. Thirdly finally, the Amadori product could be tilted such that a complexation mode results as depicted in Figure 2C. The latter binding mode suggests that binding of D-*manno*-configured Amadori rearrangement products within the FimH CRD might be possible and that Amadori products could indeed function as antagonists of natural FimH.

### Docking of Amadori products **9** and **10** into the carbohydrate binding site of FimH

In order to visualise the complexation of Amadori rearrangement products **9** and **10**, respectively, inside the binding pocket of FimH, flexible ligand docking studies were performed using the program Glide [21–24] as implemented in the Schrödinger program package (cf. Supporting Information File 1). For these studies we utilized the so-called open gate crystal structure of FimH [10]. Here, the tyrosine gate that is formed by the side chains of Y48 and Y137 at the entrance of the CRD, has an open conformation. Prior to docking, energies of the Amadori ligands were minimised with the program MacroModel [25] and afterwards 23 different conformers of **9** and 20 conformers of **10**, respectively, were generated with ConfGen [26–27] by using default settings. Next, these conformers were docked holding the FimH CRD fixed whereas conformational changes were allowed for the docked ligands under the influence of the force field. The resulting docking scores were calculated with the SP (single precision) scoring function and correlated with the binding affinity of the ligand for the FimH CRD. More negative scores indicate higher binding affinity than less negative values (Table 1).

**Table 1:** Docking scoring values of the most stable conformers complexed by FimH (open gate structure PDB 1KLF) of MeMan (**1**) in comparison with Amadori rearrangement products.

Compound	Scoring value

MeMan (**1**)	−6.6
**9**	−4.2
**10**	−5.7

According to this docking procedure, Amadori products **9** and **10** have similar scores, which lie in the range of that for MeMan (**1**). A somewhat weaker complexation is predicted for **9** and **10** than for **1**. We had expected **10** to score clearly better than **9**, owing to the possibility of π–π interactions between the phenyl substituent in **10** and the tyrosine gate at the entrance of the FimH CRD. However, this seems not to be the case.

**Figure 3:**
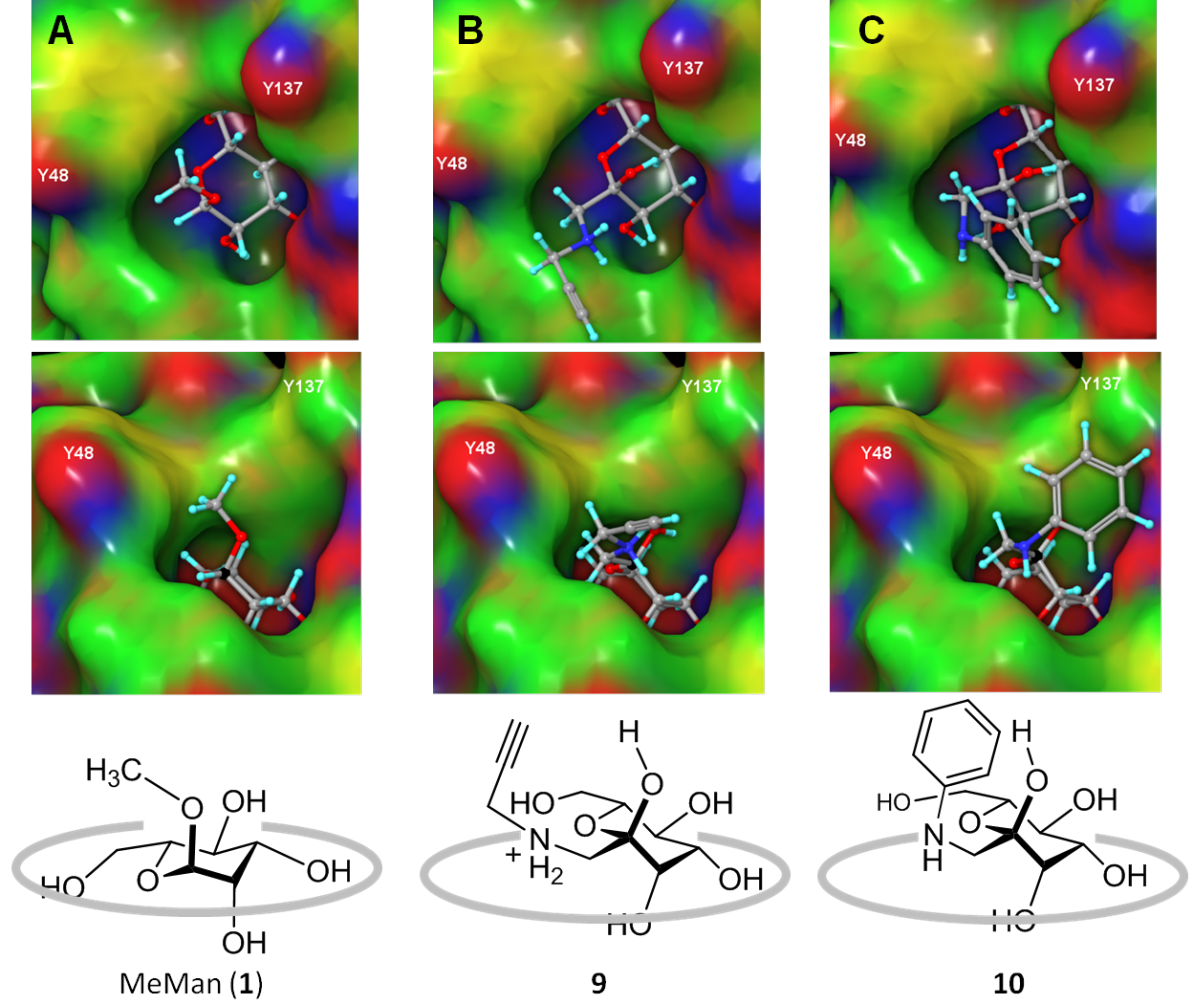
Partial charge coloured Connolly descriptions [28–29] (negative partial charges coloured in red, positive in blue) of mannoside MeMan (**1**) (A) and the Amadori products **9** (B) and **10** (C) as complexed within the CRD of FimH (PDB 1KLF, open gate structure). Top row: view from above the CRD; middle row: side view, with respective anomeric carbon atoms highlighted in black; bottom row cartoons (not to scale) correspond to side views and are drawn to illustrate that Amadori products **9** and **10** are lifted from the carbohydrate binding site resulting in diminished affinity.

We took a closer look at the docking results by comparing top scoring conformations of the different ligands (Figure 3). No difference between complexation of the Amadori products **9** and **10** and MeMan (**1**) can be seen when inspected from above the CRD. However, the side view clearly shows that the Amadori products are tilted in comparison to MeMan and somewhat lifted from the binding site (Figure 3B and C). When the respective anomeric centres are taken as a reference, **9** is lifted by 0.5 Å and **10** by 0.7 Å in comparison with complexed MeMan. The tilting effect apparently also prevents effective π–π interactions between the FimH tyrosine gate and Amadori product **10**.

**Figure 4:**
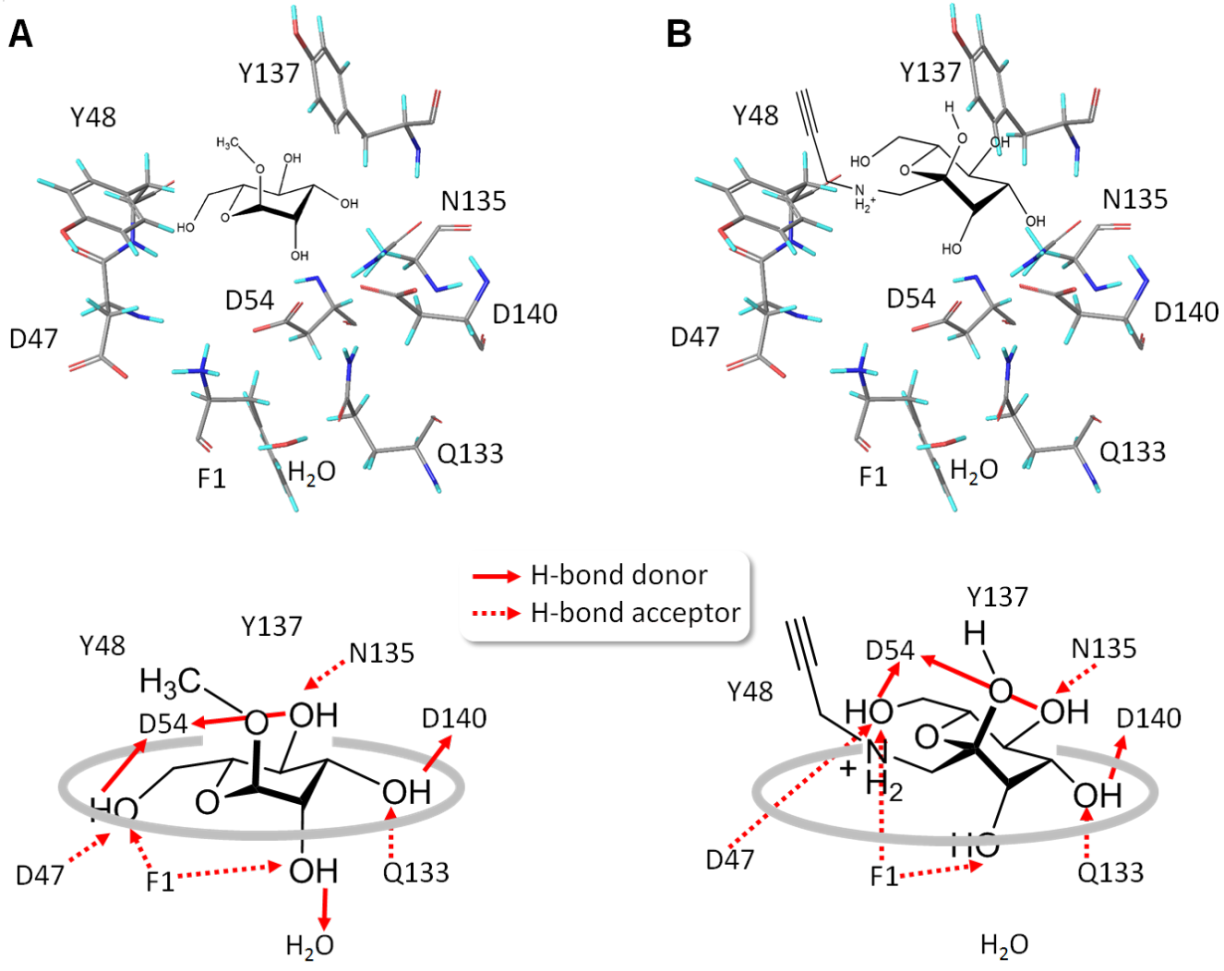
Comparison of mannosides as complexed within the CRD of FimH (PDB 1KLF). A: MeMan (**1**); B: Amadori product **9**. The top graphics show the result of the simulated 3D-arrangement of amino acid residues of the FimH CRD with docked 2D-cartoons of MeMan (**1**, left) and the Amadori product **9** (right). The bottom cartoons are deduced from the docking result illustrating the predicted hydrogen bond network between amino acid side chains and the sugar hydroxy groups (not to scale).

The effect of tilting of Amadori products **9** and **10** upon FimH complexation can also be analyzed by comparison of hydrogen bonding in the complex. Close inspection of the H-bond network reveals that the average length of H-bonds established with **9** and **10**, respectively, is higher and thus the formed H-bonds are weaker than in the case of MeMan complexation. In addition, **9** and **10** cannot interact with the water molecule that is conserved in the FimH binding site (Figure 4).

### Biological testing of Amadori products **9** and **10**

To check the predictions made by molecular docking, inhibition–adhesion studies using type 1-fimbriated fluorescent *E. coli* were performed [30]. Accordingly, the *manno*-configured glycosides **9** and **10** were used as inhibitors of FimH-mediated bacterial adhesion to mannan employing a microtiter plate format and GFP-transfected *E. coli* (pPKL1162). Serial dilutions of rearrangement products **9** and **10** in buffer were used to deliver sigmoidal inhibition curves from which IC_50_ values for both inhibitors were deduced (cf. Supporting Information File 1). The IC_50_ value of an inhibitor is the concentration at which 50% of bacterial adhesion is prevented. All assays were performed with MeMan (**1**) tested in parallel on the same plate. This allows to correlate the inhibitory potencies of **9** and **10** to that of MeMan (**1**) and report so-called relative inhibitory potencies (RIP values). This procedure allows to compare inhibitors even when they were not tested on the same plate. The results from the adhesion–inhibition assays are listed in Table 2. Both Amadori products **9** and **10** showed a lower inhibitory power than MeMan (**1**, IC_50_ ≡ 1). Thus, they have to be regarded as weak ligands for FimH. Unexpectedly, the propynyl derivative **9** has a slightly higher inhibitory power than the Amadori product **10**, having a phenyl-containing aglycone. This again shows, that the tilted complexation mode apparently compromises the possibility of favorable π–π interaction.

**Table 2:** Inhibition of bacterial adhesion (*E. coli*) to a mannan-coated surface. The inhibitory potencies of Amadori rearrangement products are compared to the standard inhibitor MeMan (**1**).^a^

	**9**	**10**

IC_50_ ± SD (mM)	7.625 ± 1.146	10.811 ± 1.470
RIP (MeMan, **1**)	0.41	0.16

^a^SD: standard deviation (from one assay); RIP: relative inhibitory potency referenced to MeMan (**1**, tested on the same microtiter plate).

## Conclusion

The Amadori rearrangement has the potential as a straight forward ligation method for conjugation of unprotected sugars and amines, when applied to suitable sugar substrates. Herein, we evaluated this synthetic method for the preparation of ligands for the α-D-mannose-specific type 1-fimbrial bacterial lectin FimH. The synthesis of heptopyranose **8** as a starting material for *manno*-configured *C*-glycosyl-type hexoses via the Amadori rearrangement was reported. We have employed propargylamine and aniline to prepare **9** and **10**, respectively. They carry an anomeric hydroxy group positioned to the α-face of the sugar ring and a rather bulky β-positioned alkyl/aryl aminomethyl group at the anomeric centre. Molecular docking of both Amadori products, **9** and **10**, into FimH suggested a reasonable binding mode, however in biological testing **9** and **10** showed an approx. 0.4 and 0.2 fold weaker potency as inhibitors of FimH-mediated bacterial adhesion than MeMan (**1**). This can be explained by the tilted fashion in which Amadori products are complexed by FimH. They are lifted from the bottom of the CRD and this results in compromised H-bonding and weak affinity.

We learn from this interdisciplinary study that it is critical to utilize the Amadori rearrangement for the synthesis of FimH ligands because it delivers products with a limited fit for this lectin. FimH complexation of D-*manno*-configured Amadori products is challenged by the steric requirements of the *C*-glycosidic aglycone. At the same time we have characterized FimH binding of a novel ligand type that encourages further development, driven by the simple synthetic availability of this type of mannoside.

## Experimental

### Materials and general methods

All chemicals were purchased from Sigma-Aldrich and used without further purification. Moisture-sensitive reactions were carried out under nitrogen in dry glassware. ^1^H and ^13^C NMR spectra were recorded on Bruker DRX-500 and AV-600 spectrometers at 300 K and 500.13 and 125.75 MHz, respectively. Chemical shifts are reported relative to internal tetramethylsilane (δ = 0.00 ppm) or D_2_O (δ = 4.76 ppm). Full assignment of the peaks was achieved with the aid of 2D NMR techniques (^1^H,^1^H-COSY and ^1^H,^13^C-HSQC). ESI mass spectra were recorded on an Esquire-LC instrument from Bruker Daltonics. Optical rotations were measured with a Perkin-Elmer 341 polarimeter (sodium D-line: 589 nm, length of cell: 1 dm, temp.: 20 °C) in the solvents indicated. Thin-layer chromatography was performed on precoated silica gel plates on aluminum 60 F254 (E. Merck 5554). Detection was effected by UV and/or charring with 10% sulfuric acid in EtOH and/or with ceric ammonium molybdate (100 g ammonium molybdate/8 g ceric sulfate in 1 L 10% H_2_SO_4_) followed by heat treatment at ≈180 °C. Flash chromatography was performed on silica gel 60 (0.035–0.070 mm, 60 A, Acros Organics 24036) using distilled solvents. For biological testing, black MaxiSorp™ plates were used from Nunc™ (Thermo Scientific™). Bacterial adhesion studies were performed according to the literature [30], using a Tecan infinite^®^ 200 multifunction microplate reader. The band pass filters’ wavelength for excitation was 485 nm and 535 nm for emission.

**2,5-Di-*O*-acetyl-3,4:6,7-di-*O*-isopropylidene-D-*glycero*-D-*galacto/D-talo*-heptopyranose (7a**, **7b):** To a solution of a C-3 diastereomeric mixture of protected oct-1-enitol derivative **6** [16–17] (4.0 g, 11 mmol) in a solvent mixture of CH_2_Cl_2_/MeOH (80 mL, 1:1 v/v), NaOAc (2.4 g, 30 mmol, 2.8 equiv) was added. This reaction mixture was treated with ozone at −50 °C for 6 h. After TLC (Cy/EtOAc, 1:1 v/v) confirmed complete consumption of the starting material, nitrogen was bubbled through the reaction mixture for 15 min and the solution was allowed to reach room temperature, followed by addition of Me_2_S (8.0 mL, 0.11 mol, 10 equiv) and stirring at rt for 45 min. The solvents were removed under reduced pressure and the obtained C-2 diastereomeric mixture of protected aldoheptoses **7a** and **7b** was used for the next step without further purification. The NMR data of the crude material confirmed signals in the expected regions.

**D-*glycero*-D-*galacto*/D-*talo*-heptopyranose (8a**, **8b):** To a solution of a C-2-epimeric mixture of compounds **7a** and **7b** (8.55 g, containing Me_2_S) in MeOH (70 mL), a solution of NaOMe (1.0 M in MeOH) was added dropwise at rt until the pH of 10 was reached and the reaction mixture was stirred at rt for 2 h until TLC (Cy/EtOAc, 1:2 v/v) showed complete consumption of the starting material. The reaction mixture was neutralized by addition of ion exchange resin (Amberlite IR 120 H^+^, washed with MeOH). The resin was filtered off, the filtrate was concentrated under reduced pressure and the crude product was purified by column chromatography (Cy/EtOAc, 4:1 v/v) to obtain a mixture of isopropylidene-protected D-*galacto*/D-*talo*-heptopyranose (3.08 g, 10.6 mmol) in 99% overall yield starting from compound **6**. The NMR data are in accordance with those reported [19]. To a solution of 3,4:6,7-di-*O*-isopropylidene-protected heptose (2.50 g, 8.61 mmol) in a mixture of MeCN/H_2_O (50 mL, 1:1 v/v,), acidic ion exchange resin (Amberlite IR 120 H^+^, washed with H_2_O) was added until a pH of 2 was reached and the reaction mixture was stirred at 40 °C for 1 h. After TLC (CHCl_3_/MeOH/concd. NH_4_OH, 1/2/1 v/v/v) showed complete consumption of the starting material, the resin was filtered off and the filtrate was concentrated under reduced pressure. Column chromatography (CHCl_3_/MeOH 10:1 v/v) gave D-*glycero*-D-*galacto*/D-*talo-*heptopyranoses **8a** and **8b** (1.55 g, 7.39 mmol) in a yield of 86%. The NMR data are in accordance with those reported [2–3].

**1-(*N*-Propargyl)amino-1-deoxy-α-D-*manno*-hept-2-ulose (9):** To a solution of D-*glycero*-D-*galacto*/D-*talo*-heptose **8a** and **8b** (467 mg, 2.22 mmol) in a mixture of EtOH (7 mL), 1,4-dioxane (1 mL) and water (2 drops), propargylamine (142 µL, 2.22 mmol, 1.0 equiv) and acetic acid (127 µL, 2.22 µmol, 1.0 equiv) were added and the reaction mixture was stirred at 70 °C for two days. Complete consumption of the starting material was indicated by TLC (CHCl_3_/MeOH/NH_4_OH, 1:2:1 v/v/v). The solvents were removed under reduced pressure and subsequent column chromatography (CHCl_3_/MeOH, 8:1 v/v containing 1% of concd. NH_4_OH) gave 1-propargylamino-modified ketose **9** (420 mg, 1.70 mmol) in a yield of 77%. [α]_D_ +13.2 (*c* 2.5, MeOH); ^1^H NMR (500 MHz, MeOH-*d*_4_) δ 3.84 (dd, 1H, H-4), 3.82 (dd, *J*_7,6_ = 2.2 Hz, 1H, H-7), 3.80 (d, *J*_3,4_ = 3.3 Hz, 1H, H-3), 3.74 (dd, *J*_7,7’_ = 11.5 Hz, *J*_7’,6_ = 5.5 Hz, 1H, H-7’), 3.72–3.69 (m, 1H, H-6), 3.62 (dd, *J*_4,5_ = 9.4 Hz, *J*_5,6_ = 9.5 Hz, 1H, H-5), 3.58 (d, 2H, H-8), 3.10 (d, *J*_1,1’_ = 12.3 Hz, 1H, H-1), 2.96 (d, 1H, H-1’), 2.76 (t, 1H, H-10); ^13^C NMR (125 MHz, MeOH-*d*_4_) δ 97.4 (C-2), 80.3 (C-9), 74.8 (2C, C-3, C-6), 74.5 (C-10), 72.9 (C-4), 68.2 (C-5), 62.8 (C-7), 55.4 (C-1), 38.6 (C-8); ESIMS (*m/z*): calcd for [C_10_H_17_NO_6_ + H]^+^, 248.1134; found, 248.113 [M + H]^+^.

**1-(*N*-Phenyl)amino-1-deoxy-α-D-*manno*-hept-2-ulose (10):** To a solution of D-*glycero*-D-*galacto*/D-*talo-*heptopyranoses **8a** and **8b** (110 mg, 523 µmol) in a mixture of EtOH (1 mL), 1,4-dioxane (0.2 mL) and water (2 drops), aniline (47.8 µL, 523 µmol, 1.0 equiv) and acetic acid (30.0 µL, 523 µmol, 1.0 equiv) were added and the reaction mixture was stirred at 70 °C for 48 h. Complete consumption of the starting material was indicated by TLC (CHCl_3_/MeOH/NH_4_OH, 1:2:1 v/v/v). The solvents were removed under reduced pressure and subsequent column chromatography (CHCl_3_/MeOH, 8:1 v/v containing 1% of concd. NH_4_OH) gave 1-phenylamino ketose **10** (35.0 mg, 123 µmol) in a yield of 24%. [α]_D_ +21.5 (*c* 0.76, MeOH); ^1^H NMR (500 MHz, MeOH-*d*_4_) δ 7.11 (dd, 2H, phenyl), 6.75 (d, 2H, phenyl), 6.65 (dd, 1H, phenyl), 3.90 (dd, *J*_3,4_ = 3.3 Hz, *J*_4,5_ = 9.4 Hz, 1H, H-4), 3.85 (d, 1H, H-3), 3.87–3.83 (m, 1H, H-7), 3.78–3.76 (m, 1H, H-6), 3.75 (dd, *J*_6,7’_ = 5.3 Hz, *J*_7,7’_ = 13.7 Hz, 1H, H-7’), 3.63 (dd, *J*_5,6_ = 9.5 Hz, 1H, H-5), 3.43 (d, *J*_1,1’_ = 12.7 Hz, 1H, H-1), 3.27 (d,1H, H-1’); ^13^C NMR (125 MHz, MeOH-*d*_4_) δ 150.2, 130.0, 118.7, 114.8 (6C, phenyl), 98.9 (C-2), 74.9 (C-6), 73.3 (C-3), 72.9 (C-4), 68.7 (C-5), 63.0 (C-7), 51.4 (C-1). ESIMS (*m/z*): calcd for [C_13_H_19_NO_6_ + H]^+^, 286.1291; found, 286.129 [M + H]^+^.

## Supporting Information

File 1 NMR spectra, boiassays and molecular docking.
