# Supplementary material for: Are D-manno-configured Amadori products ligands of the bacterial lectin FimH?
Source: Beilstein J Org Chem. 2015 Jun 30;11:1096–104. doi: 10.3762/bjoc.11.123 (PMC4505182; doi:10.3762/bjoc.11.123)
Supplement: File 1 — NMR spectra, boiassays and molecular docking. [file Beilstein_J_Org_Chem-11-1096-s001.pdf]

Supporting Information

for

Are D-*manno*-configured Amadori products ligands of the bacterial lectin FimH?

Tobias-Elias Gloe[‡1], Insa Stamer[‡1], Cornelia Hojnik[‡2], Tanja M. Wrodnigg*[2], Thisbe K. Lindhorst*[1]

Address: [1]Christiana Albertina University of Kiel, Otto Diels Institute of Organic Chemistry, Otto-Hahn-Platz 3/4, D-24118 Kiel, Germany, Fax: +49 431 8807410, and [2]Glycogroup, Institute of Organic Chemistry, Technical University Graz, Stremayrgasse 9, A-8010 Graz, Austria

Email: Tanja M. Wrodnigg* - t.wrodnigg@tugraz.at; Thisbe K. Lindhorst - tklind@oc.uni-kiel.de

*Corresponding author

[‡]These authors have contributed equally.

NMR spectra, bioassays and molecular docking

Table of contents

1. **^{1}H and ^{13}C NMR spectra of 9 and 10** S2

2. **Bioassays** S4

3. **Docking studies** S6

4. **References** S8

1. ^{1}H and ^{13}C NMR spectra

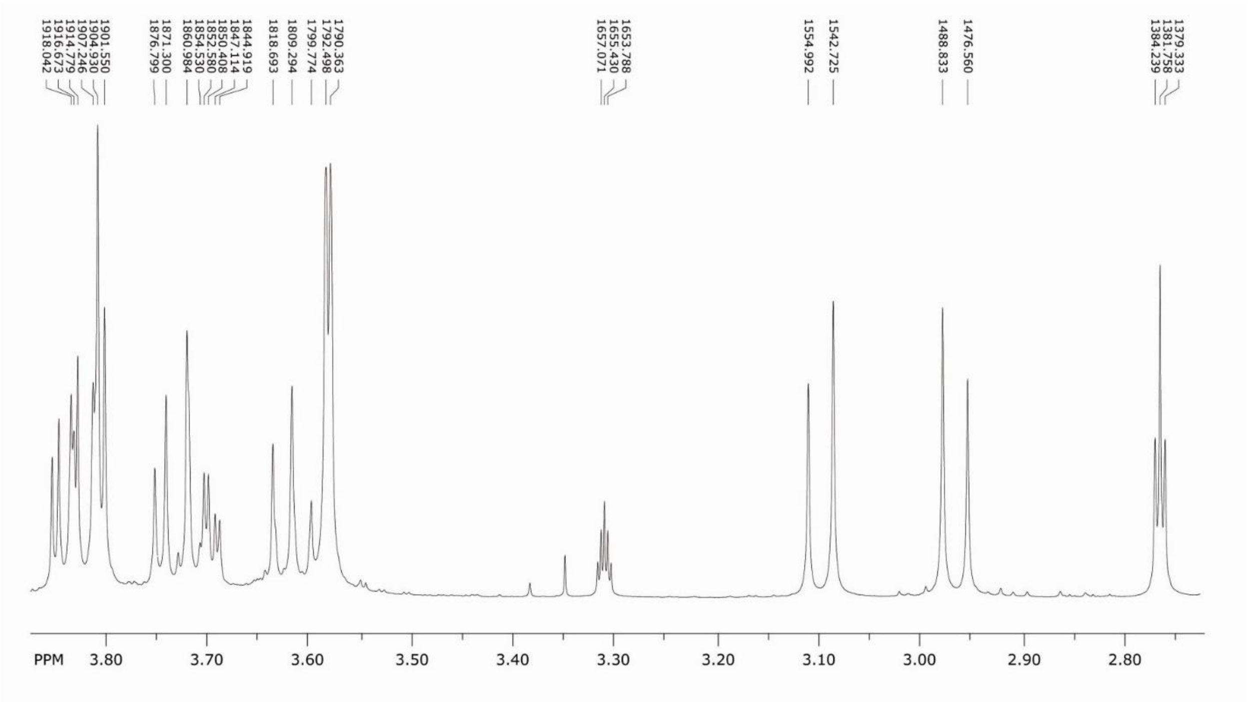

Figure S1: ^{1}H NMR spectrum of **9** in MeOD-d_4 (500 MHz).

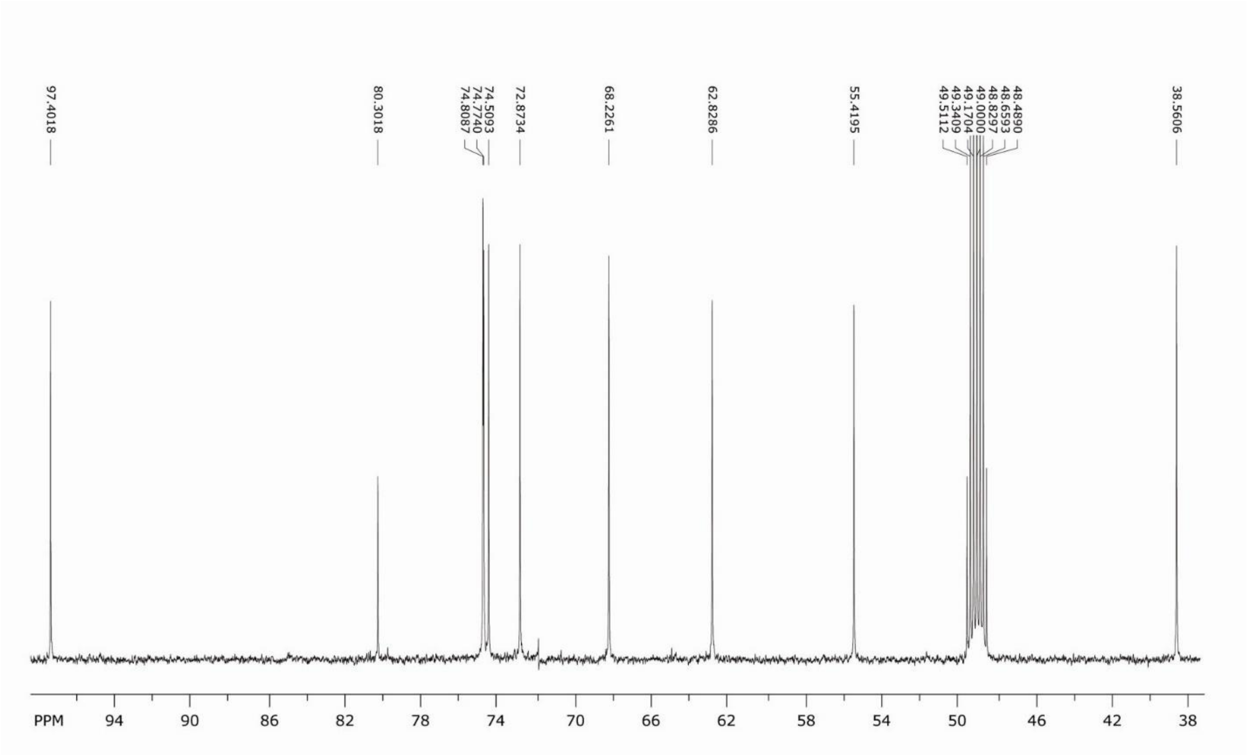

Figure S2: ^{13}C NMR spectrum of **9** in MeOD-d_4 (125 MHz).

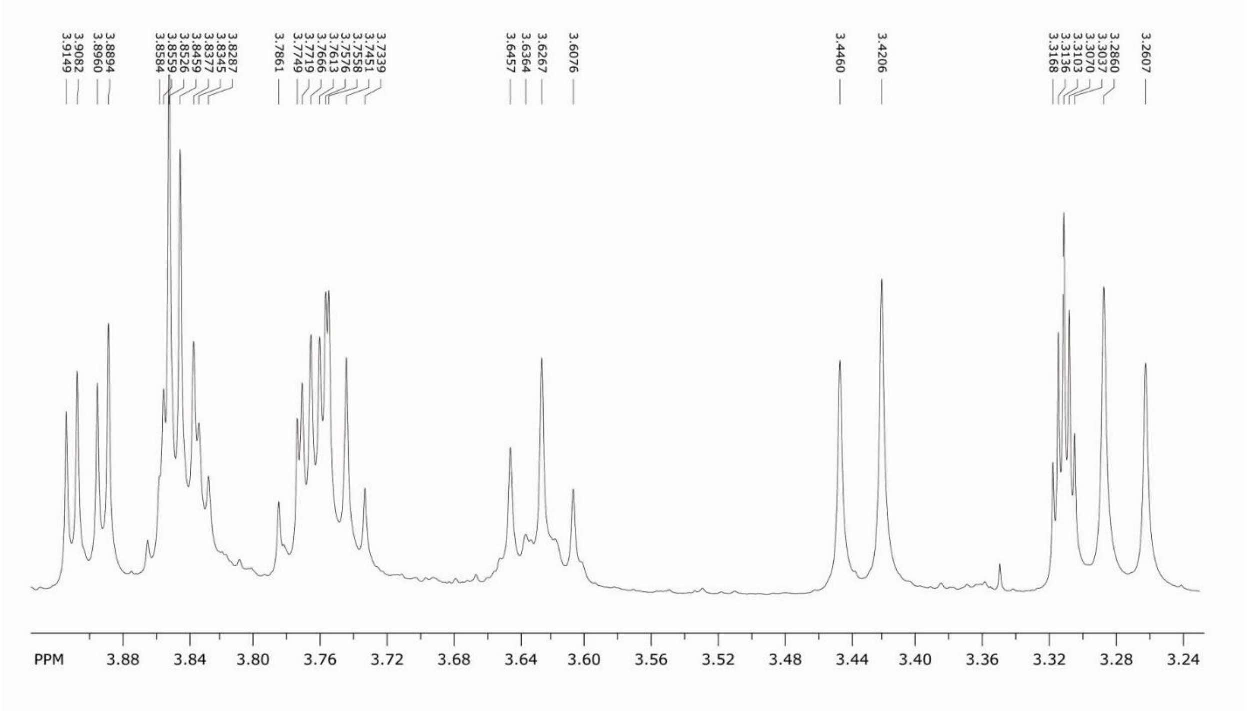

Figure S3: ^{1}H NMR spectrum of **10** in MeOD-d_4 (500 MHz).

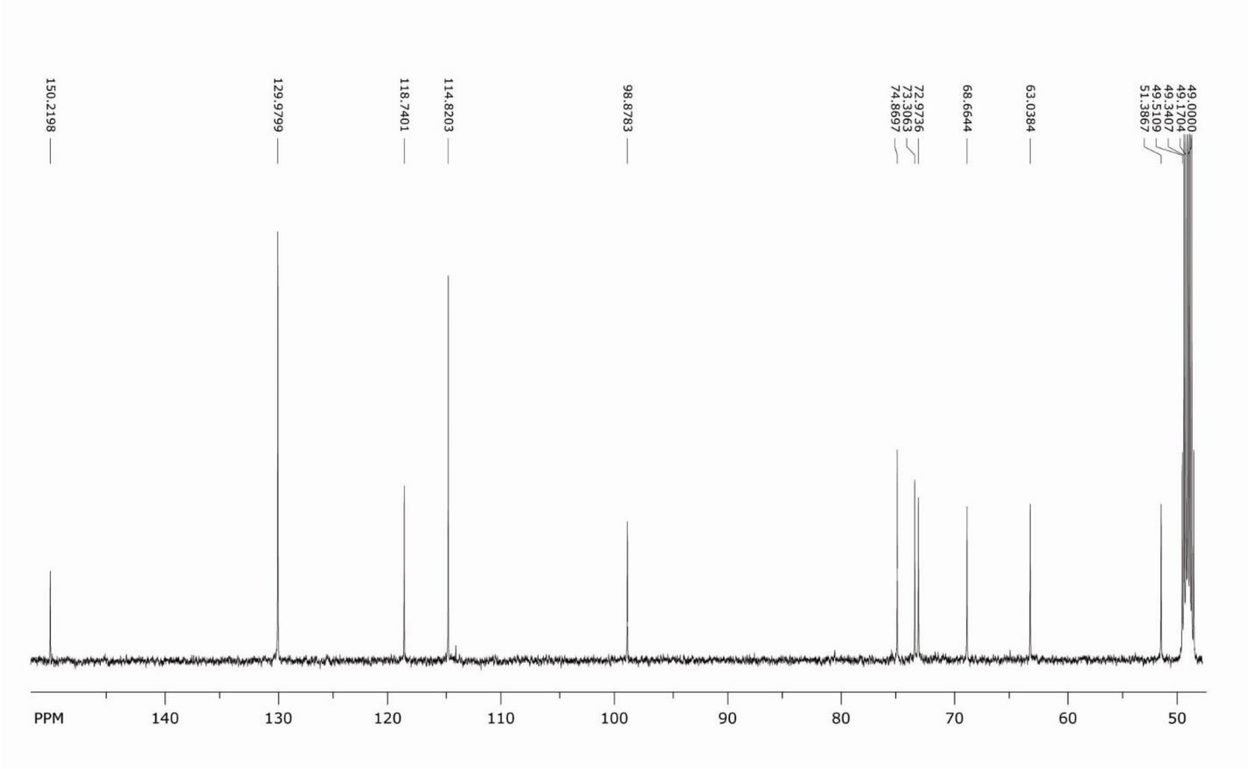

Figure S4: ^{13}C NMR spectrum of **10** in MeOD-d_4 (125 MHz).

2. Bioassays

Media and buffer solutions: Carbonate buffer solution (pH 9.6): sodium carbonate (1.59 g) and sodium hydrogen carbonate (2.52 g) were dissolved in distilled deionized water (1.00 L). PBS buffer solution (pH 7.2): sodium chloride (8.00 g), potassium chloride (200 mg), sodium hydrogen phosphate dihydrate (1.44 g) and potassium dihydrogen phosphate (200 mg) were dissolved in distilled deionized water (1.00 L). PBST buffer solution (pH 7.2): PBS buffer + Tween® 20 (0.05% v/v). LB medium: tryptone (10.0 g), sodium chloride (10.0 g) and yeast extract (5.00 g) were dissolved in distilled deionized water (1.00 L); after autoclavation chloramphenicol (50.0 mg) and ampicillin (100 mg) were added. The buffer pH values were adjusted with aqueous 0.1 M HCl or 0.1 M NaOH solution.

Cultivation of bacteria: *E. coli* bacteria (strain pPKL1162) [1,2] were cultured from a frozen stock in LB medium and incubated overnight at 37 °C. After centrifugation and washing twice with PBS buffer (2.00 mL), the bacteria pellet was suspended in PBS buffer and the suspension was adjusted to OD_{600} = 0.4 (2 mg/mL) with PBS.

GFP assay: The published assay [3] was adapted and modified as follows: Black 96-well microtiter plates (Nunc, MaxiSorp) plates were treated with a solution of mannan from *Saccharomyces cerevisiae* (1.2 mg/mL in carbonate buffer, 120 µL/well) and desiccated overnight at 37 °C. After washing for three times with PBST (150 µL/well), the wells were blocked with PVA (1% in PBS, 120 µL/well) for 4 h at 4 °C. Subsequently, the plates were washed twice with PBST (150 µL/well) and once with PBS (150 µL/well). Solutions of Amadori compounds **9** and **10** as well as MeMan (**1**) were prepared (200 mM in PBS) and serial dilutions of each solution added to the mannan-coated plates (50 µl/well). Then the bacterial suspension (OD_{600} = 0.4, 50 µL/well) was added and the plates were incubated for 1 h at 37 °C and 100 rpm. After washing twice with PBS (150 µL), the wells were filled with PBS (100 µL/well) and the fluorescence intensity (485 nm/535 nm) was determined.

Each compound was tested at least in triplicate and in parallel with the standard inhibitor MeMan (**1**) on the same plate.

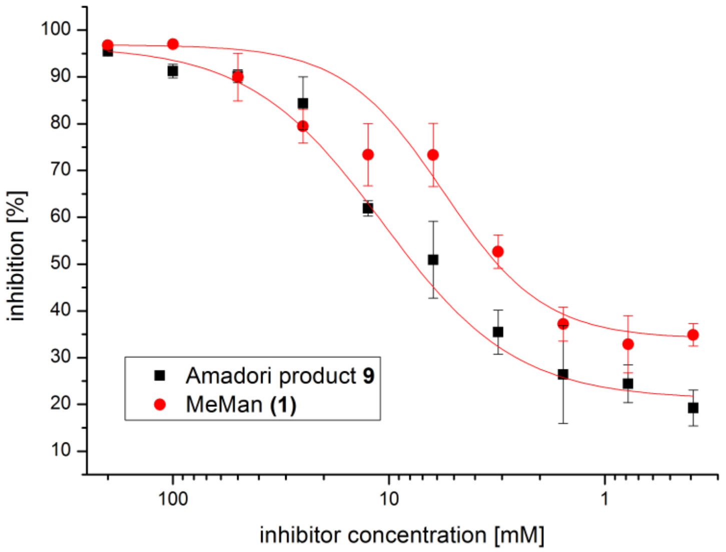

Figure S5: Inhibition curves obtained with Amadori product **9** from inhibition of type 1 fimbriae-mediated bacterial adhesion to mannan. MeMan (**1**) was tested on the same microtiter plate. The sigmoidal concentration–response curves were fitted by non-linear regression. Error bars are standard deviations from multiple (at least three) testing results on one plate.

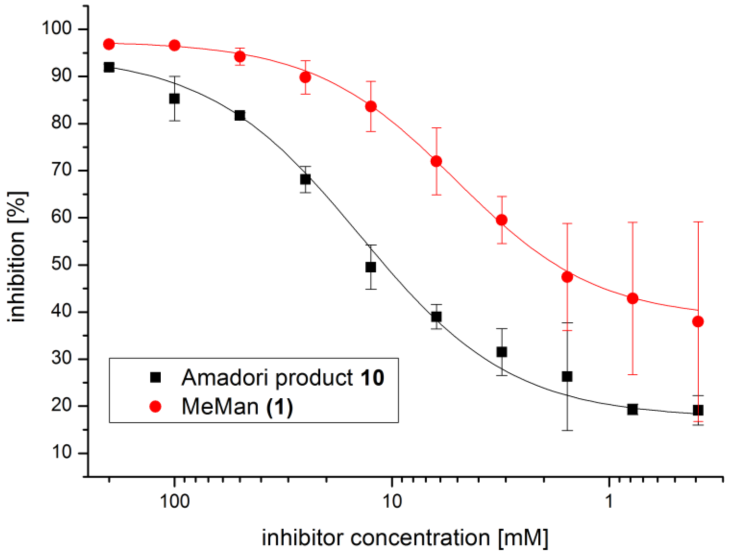

Figure S6: Inhibition curves obtained with Amadori product **10** from inhibition of type 1 fimbriae-mediated bacterial adhesion to mannan. MeMan (**1**) was tested on the same microtiter plate. The sigmoidal concentration–response curves were fitted by non-linear regression. Error bars are standard deviations from multiple (at least three) testing results on one plate.

3. Docking studies

Computer-aided docking was performed using flexible ligand docking with Glide [4-7] as implemented in the Schrödinger program package. Docking was based on the open gate crystal structure (PDB code 1KLF) of FimH [8]. The structures were minimized with the program MacroModel [9] by using default settings resulting in 23 different conformers of **9**, 20 conformers of **10** and 9 conformers of MeMan (**1**). The conformers were generated with ConfGen [10,11] and are listed in Tables S1–S3 with the respective scoring values.

Table S1: Scoring values for docking of Amadori product **9** into the open gate crystal structure of FimH.

no.	docking score	glide gscore	glide evdw	glide ecoul	glide lipo	glide hbond	glide metal	glide rewards	glide erotb	glide esite
1	-4.230	-5.423	-11.659	-30.573	-0.081	0.000	0.000	-1.716	0.992	-0.034
2	-4.229	-5.422	-11.356	-31.026	-0.096	0.000	0.000	-1.716	0.992	-0.033
3	-4.228	-5.421	-11.633	-30.576	-0.081	0.000	0.000	-1.716	0.992	-0.034
4	-4.228	-5.421	-11.626	-30.584	-0.081	0.000	0.000	-1.716	0.992	-0.034
5	-4.228	-5.421	-11.634	-30.574	-0.081	0.000	0.000	-1.716	0.992	-0.034
6	-4.210	-5.403	-11.044	-33.054	-0.096	0.000	0.000	-1.716	0.992	-0.030
7	-4.210	-5.403	-11.042	-33.066	-0.096	0.000	0.000	-1.716	0.992	-0.030
8	-4.210	-5.403	-11.038	-33.054	-0.096	0.000	0.000	-1.716	0.992	-0.030
9	-4.209	-5.402	-11.026	-33.080	-0.096	0.000	0.000	-1.716	0.992	-0.030
10	-4.209	-5.402	-11.013	-33.092	-0.096	0.000	0.000	-1.716	0.992	-0.030
11	-4.208	-5.401	-10.999	-33.094	-0.097	0.000	0.000	-1.716	0.992	-0.030
12	-4.208	-5.401	-10.994	-33.080	-0.097	0.000	0.000	-1.716	0.992	-0.030
13	-4.207	-5.400	-11.194	-30.605	-0.081	0.000	0.000	-1.716	0.992	-0.035
14	-4.207	-5.400	-11.192	-30.609	-0.081	0.000	0.000	-1.716	0.992	-0.035
15	-4.206	-5.399	-11.187	-30.614	-0.081	0.000	0.000	-1.716	0.992	-0.035
16	-4.206	-5.399	-11.183	-30.617	-0.081	0.000	0.000	-1.716	0.992	-0.035
17	-4.206	-5.399	-11.197	-30.594	-0.080	0.000	0.000	-1.716	0.992	-0.035
18	-4.187	-5.380	-10.574	-32.942	-0.097	0.000	0.000	-1.716	0.992	-0.030
19	-4.187	-5.380	-10.567	-32.944	-0.097	0.000	0.000	-1.716	0.992	-0.030
20	-4.186	-5.379	-10.546	-32.941	-0.097	0.000	0.000	-1.716	0.992	-0.030
21	-4.186	-5.379	-10.541	-32.944	-0.097	0.000	0.000	-1.716	0.992	-0.030
22	-4.167	-5.360	-10.165	-33.077	-0.095	0.000	0.000	-1.716	0.992	-0.032
23	-4.148	-5.341	-10.483	-33.017	-0.062	0.000	0.000	-1.716	0.992	-0.030

Table S2: Scoring values for docking of Amadori product **10** into the open gate crystal structure of FimH.

no.	docking score	glide gscore	glide evdw	glide ecoul	glide lipo	glide hbond	glide metal	glide rewards	glide erotb	glide esite
1	-5.693	-5.693	-15.656	-29.241	-0.041	0.000	0.000	-1.464	0.624	-0.029
2	-5.678	-5.678	-15.284	-29.690	-0.046	0.000	0.000	-1.464	0.624	-0.028
3	-5.671	-5.671	-15.162	-29.707	-0.045	0.000	0.000	-1.464	0.624	-0.028
4	-5.656	-5.656	-13.640	-31.348	-0.103	0.000	0.000	-1.464	0.624	-0.032
5	-5.653	-5.653	-14.811	-29.556	-0.045	0.000	0.000	-1.464	0.624	-0.028
6	-5.650	-5.650	-13.822	-33.156	-0.090	0.000	0.000	-1.464	0.624	-0.029
7	-5.645	-5.645	-13.374	-31.571	-0.104	0.000	0.000	-1.464	0.624	-0.032
8	-5.643	-5.643	-14.593	-30.063	-0.046	0.000	0.000	-1.464	0.624	-0.028
9	-5.625	-5.625	-13.506	-33.189	-0.089	0.000	0.000	-1.464	0.624	-0.021
10	-5.620	-5.620	-14.103	-30.763	-0.048	0.000	0.000	-1.464	0.624	-0.028
11	-5.619	-5.619	-13.139	-31.763	-0.093	0.000	0.000	-1.464	0.624	-0.029
12	-5.615	-5.615	-13.053	-31.784	-0.094	0.000	0.000	-1.464	0.624	-0.029
13	-5.615	-5.615	-13.050	-31.767	-0.094	0.000	0.000	-1.464	0.624	-0.029
14	-5.615	-5.615	-13.041	-31.787	-0.094	0.000	0.000	-1.464	0.624	-0.029
15	-5.614	-5.614	-13.035	-31.779	-0.094	0.000	0.000	-1.464	0.624	-0.029
16	-5.573	-5.573	-12.708	-32.117	-0.066	0.000	0.000	-1.464	0.624	-0.032
17	-5.573	-5.573	-12.709	-32.109	-0.066	0.000	0.000	-1.464	0.624	-0.032
18	-5.572	-5.572	-12.702	-32.123	-0.066	0.000	0.000	-1.464	0.624	-0.032
19	-5.571	-5.571	-12.682	-32.141	-0.066	0.000	0.000	-1.464	0.624	-0.032
20	-5.558	-5.558	-12.334	-32.105	-0.071	0.000	0.000	-1.464	0.624	-0.031

Table S3: Scoring values for docking of MeMan (**1**) into the open gate crystal structure of FimH.

no.	docking score	glide gscore	glide evdw	glide ecoul	glide lipo	glide hbond	glide metal	glide rewards	glide erotb	glide esite
1	-6.567	-6.567	-12.463	-28.026	-0.145	0.000	0.000	-2.085	0.300	-0.014
2	-6.564	-6.564	-12.418	-28.059	-0.144	0.000	0.000	-2.085	0.300	-0.014
3	-6.528	-6.528	-12.111	-28.445	-0.124	0.000	0.000	-2.085	0.300	-0.014
4	-6.525	-6.525	-12.059	-28.484	-0.123	0.000	0.000	-2.085	0.300	-0.014
5	-6.486	-6.486	-11.170	-29.242	-0.120	0.000	0.000	-2.085	0.300	-0.023
6	-6.484	-6.484	-11.142	-29.269	-0.119	0.000	0.000	-2.085	0.300	-0.022
7	-6.483	-6.483	-11.144	-29.281	-0.119	0.000	0.000	-2.085	0.300	-0.022
8	-6.482	-6.482	-11.125	-29.293	-0.118	0.000	0.000	-2.085	0.300	-0.022
9	-6.203	-6.203	-6.927	-33.084	-0.051	0.000	0.000	-2.085	0.300	-0.021

4. References

[1] Reisner, A.; Haagensen, J. A. J.; Schembri, M. A.; Zechner, E. L.; Molin, S., *Mol. Microbiol.* **2003**, *48*, 933-946.

[2] The GFP-tagged strain pPKL1162 was constructed in the Klemm group by introduction of the plasmid pPKL174 into strain SAR18; pPKL174 contains the fim gene cluster required for type 1 fimbriae assembly and expression. The chromosome of strain SAR18 from the Reisner group contains the GFP gene, controlled by a constitutive promoter.

[3] Hartmann, M.; Horst, A. K.; Klemm, P.; Lindhorst, T. K., *Chem. Commun.* **2010**, *46*, 330-332.

[4] Friesner, R. A.; Murphy, R. B.; Repasky, M. P.; Frye, L. L.; Greenwood, J. R.; Halgren, T. A.; Sanschagrin, P. C.; Mainz, D. T., *J. Med. Chem.* **2006**, *49*, 6177-6196.

[5] Halgren, T. A.; Murphy, R. B.; Friesner, R. A.; Beard, H. S.; Frye, L. L.; Pollard, W. T.; Banks, J. L.; *J. Med. Chem.* **2004**, *47*, 1750-1759.

[6] Friesner, R. A.; Banks, J. L.; Murphy, R. B.; Halgren, T. A.; Klicic, J. J.; Mainz, D. T.; Repasky, M. P.; Knoll, E. H.; Shelley, M.; Perry, J. K.; Shaw, D. E.; Francis, P.; Shenkin, P. S., *J. Med. Chem.* **2004**, *47*, 1739-1749.

[7] *Small-Molecule Drug Discovery Suite 2013-3: Glide*, version 6.1; Schrödinger, LLC: New York, NY, **2013**.

[8] Hung, C. S.; Bouckaert, J.; Hung, D.; Pinkner, J.; Widberg, C.; DeFusco, A.; Auguste, C. G.; Strouse, R.; Langermann, S.; Waksman, G.; Hultgren, S. J., *Mol. Microbiol.* **2002**, *44*, 903-15.

[9] *Schrödinger Release 2013-3: MacroModel*, version 10.2; Schrödinger, LLC: New York, NY, **2013**.

[10] Watts, K. S.; Dalal, P.; Murphy, R. B.; Sherman, W.; Friesner, R. A.; Shelley, J. C., *J. Chem. Inf. Model.* **2010**, *50*, 534-546.

[11] *Schrödinger Release 2013-3: ConfGen*, version 2.6; Schrödinger, LLC: New York, NY, **2013**.
